# Antibacterial Efficacy of Polysaccharide Capped Silver Nanoparticles Is Not Compromised by AcrAB-TolC Efflux Pump

**DOI:** 10.3389/fmicb.2018.00823

**Published:** 2018-05-04

**Authors:** Mitali Mishra, Satish Kumar, Rakesh K. Majhi, Luna Goswami, Chandan Goswami, Harapriya Mohapatra

**Affiliations:** ^1^School of Biological Sciences, National Institute of Science Education and Research, Homi Bhabha National Institute, Bhubaneswar, India; ^2^School of Biotechnology, Kalinga Institute of Industrial Technology, Bhubaneswar, India

**Keywords:** nanoparticles, efflux pump, AcrAB-TolC, *Enterobacter cloacae*, antibiotic resistance

## Abstract

Antibacterial therapy is of paramount importance in treatment of several acute and chronic infectious diseases caused by pathogens. Over the years extensive use and misuse of antimicrobial agents has led to emergence of multidrug resistant (MDR) and extensive drug resistant (XDR) pathogens. This drastic escalation in resistant phenotype has limited the efficacy of available therapeutic options. Thus, the need of the hour is to look for alternative therapeutic approaches to mitigate healthcare concerns caused due to MDR bacterial infections. Nanoparticles have gathered much attention as potential candidates for antibacterial therapy. Equipped with advantages of, wide spectrum bactericidal activity at very low dosage, inhibitor of biofilm formation and ease of permeability, nanoparticles have been considered as leading therapeutic candidates to curtail infections resulting from MDR bacteria. However, substrate non-specificity of efflux pumps, particularly those belonging to resistance nodulation division super family, have been reported to reduce efficacy of many potent antibacterial therapeutic drugs. Previously, we had reported antibacterial activity of polysaccharide-capped silver nanoparticles (AgNPs) toward MDR bacteria. We showed that AgNPs inhibits biofilm formation and alters expression of cytoskeletal proteins FtsZ and FtsA, with minimal cytotoxicity toward mammalian cells. In the present study, we report no reduction in antibacterial efficacy of silver nanoparticles in presence of AcrAB-TolC efflux pump proteins. Antibacterial tests were performed according to CLSI macrobroth dilution method, which revealed that both silver nanoparticles exhibited bactericidal activity at very low concentrations. Further, immunoblotting results indicated that both the nanoparticles modulate the transporter AcrB protein expression. However, expression of the membrane fusion protein AcrA did show a significant increase after exposure to AgNPs. Our results indicate that both silver nanoparticles are effective in eliminating MDR *Enterobacter cloacae* isolates and their action was not inhibited by AcrAB-TolC efflux protein expression. As such, the above nanoparticles have strong potential to be used as effective and alternate therapeutic candidates to combat MDR gram-negative Enterobacterial pathogens.

## Introduction

The drastic escalation in the proportion of multiple antibiotic resistant bacteria is recognized as a serious health care concern globally (WHO, 2014). The evolution of drug resistant microbes has challenged the success of antimicrobial agents for combating infectious diseases. The group of opportunistic pathogens, belonging to genera *Enterococcus, Staphylococcus, Klebsiella, Acinetobacter, Pseudomonas*, and *Enterobacter* (acronymed as ESKAPE) are exhibiting resistance to virtually all available antimicrobial agents (Boucher et al., 2009). Indeed, the acronym ‘ESKAPE’ is perfect choice to reflect these ‘bad bugs.’ To complicate the scenario, many of the above-mentioned bacteria are increasingly becoming responsible for spread of multidrug resistant (MDR) community acquired infections (Pendleton et al., 2013).

The *Enterobacter* spp., particularly *E. aerogenes* and *E. cloacae* are often associated with nosocomial infections like urinary tract infection (specifically catheter related), abdominal cavity/intestinal infections, wound infections, pneumonia, and septicemia (Sanders and Sanders, 1997; Fraser and Arnett, 2010). These two clinically predominant species display one or more resistance mechanisms toward antimicrobials like β-lactams, cephalosporins, aminoglycosides. Ironically, *E. aerogenes* and *E. cloacae* are also exhibiting trends of increased resistance toward the last resort antimicrobial agents *–* carbapenems and colistin (Davin-Regli and Pagès, 2015). Well-equipped with armory of antibiotic resistance strategies such as, outer membrane permeability barrier, efflux pumps and antibiotic degrading enzymes, *Enterobacter* spp. form an interesting infectious model to explore (Grimont and Grimont, 2006; Mezzatesta et al., 2012; Davin-Regli and Pagès, 2015).

The rise in antimicrobial resistance coupled with diminishing available options has necessitated searching for alternative therapeutic options (Martins and McCusker, 2016). One such lucrative direction was use of combinatorial therapy for treatment of infections caused by gram-negative pathogens, such as *Pseudomonas aeruginosa*. Typically, the combinatorial therapies for gram-negative pathogens included a β-lactam with aminoglycoside or fluoroquinolone (Tamma et al., 2012). The strategy was to extract their synergistic potential and broader spectrum of activity. But, in many clinical trials, it developed adverse effects leading to nephrotoxicity and even greater multiple antibiotic resistance (Kerantzas and Jacobs, 2017). The limitations faced by combinatorial therapies led to further improvisation and antimicrobial peptides were the more sought after. These host-defense peptides were investigated due to their antimicrobial properties against both gram-positive and gram-negative pathogens and immunomodulatory roles. However, low metabolic stability and discrepancies between *in vivo* and *in vitro* antimicrobial effectiveness limited the scope of antimicrobial peptides (Mahlapuu et al., 2016). Other interesting alternatives were more conceptual driven, such as bacteriophage therapy (Yang et al., 2014) and/or repurposing the existing drugs for better antibacterial and anti-virulence features (Rangel-Vega et al., 2015). Although both of them appeared as promising candidates, but development of such therapy at large scale remains challenging and yet to be explored.

All of the above-developed strategies faced severe obstacles in getting translated for sustainable, practical and large-scale usage, as demanded by the present situation. Much of the aforementioned limitations were circumvented by development of nanoparticle as drugs (Salata, 2004; Singh and Lillard, 2009). Over the years, nanoparticle-based approaches have gathered much reputation due to their fast and effective antibacterial properties for various pathogens (Gelperina et al., 2005; Tran and Webster, 2011). Nanoparticles endowed with wide bactericidal spectrum, ability to inhibit biofilm, increased permeability and efficacy at low dose, form a powerful weapon to combat MDR bacteria (Kim et al., 2007; Pelgrift and Friedman, 2013; Chen et al., 2014; Sanyasi et al., 2016). Review of the literature reveals that presence of efflux pumps [particularly those belonging to resistance nodulation division (RND) super family, e.g., tripartite efflux pump AcrAB-TolC] in gram-negative bacteria compromises the antibacterial efficacy of the drugs by extruding them out of the cytoplasm (Zgurskaya, 2009). Efflux of drugs reduces their intracellular concentration, leading to increased resistance in pathogens toward the drug. Further, it has been reported that substrates to be effluxed (antibacterial drugs) induce overexpression of the efflux proteins (Rosenberg et al., 2003).

Previously we had reported antibacterial activity of polysaccharide-capped silver nanoparticles (AgNPs) toward several MDR bacteria *viz. E. coli, Klebsiella* spp., *Enterobacter* spp., and *S. aureus* (Sanyasi et al., 2016). We had demonstrated AgNPs to inhibit biofilm formation and alter expression of cytoskeletal proteins FtsZ and FtsA, with minimal cytotoxicity toward mammalian cells. In the present study, we have further investigated antibacterial effects of the aforementioned AgNPs and that of a newly developed silver-metal-carbohydrate nanoparticle (Ag-MCNP) particularly on MDR *E. cloacae* isolates. Further, with the objective to consolidate our findings, we also investigated the effect of these silver nanoparticles on expression of AcrAB-TolC efflux pump proteins in MDR *E. cloacae* isolates.

## Materials and Methods

### Bacterial Strains and Media

A wild type *E. cloacae* (EspIMS6) urinary tract infection isolate from a tertiary care hospital at Bhubaneswar, India, and *E. cloacae* subsp. *cloacae* ATCC-13047 type strain were used in the present study. The bacterial isolates were identified by biochemical tests followed by 16S rRNA gene sequencing. As prescribed for Enterobacteriaceae (CLSI, 2016), *E. coli* strain ATCC 25922 was used as control during antibiotic susceptibility testing. Unless otherwise mentioned, bacterial cultures were grown on nutrient agar/broth (Hi-Media, India) and incubated at 37°C in a controlled environment shaker (New Brunswick, NJ, United States). Muller Hinton broth (MHB)/agar (MHA) medium (Hi-Media, India) was used for antibiotic susceptibility test. Following sub-culture, the strains were preserved at -80°C in 20% glycerol for further use. All experiments were performed in triplicate and repeated at least three times.

### Resistance Profiling

Antibiotic disks used in resistance profiling were obtained from Himedia, India. Each strain was tested for susceptibility to different antibiotics by disk diffusion method (Bauer et al., 1966). The diameter of the inhibition zones was interpreted following CLSI standard as proposed for Enterobacteriaceae (CLSI, 2016).

### Characterization of Silver Nanoparticles Used

#### Physical Characterization of the Nanoparticles Used

Particle size and zeta potential (ζ) of the polysaccharide capped silver nanoparticles were measured by Zetasizer Nano ZS instrument (Malvern Instruments, United Kingdom) at a constant temperature of 25 ± 1°C. The samples (0.1 mg/ml) were suspended in Milli-Q water and sonicated for 1 min. The mean hydrodynamic diameter and zeta potential for each sample was measured in triplicate and the results were measured as mean size ± SD. The surface plasmon resonance (SPR) was recorded at different time points (10–150 min) for polysaccharide-capped silver nanoparticles (AgNP) against the only carboxyl methyl tamarind (CMT) polysaccharide solution as blank.

#### Determining Hemocompatibility of Silver Nanoparticle

Hemocompatibility of polysaccharide capped silver nanoparticle was determined in terms of percent hemolysis using sheep blood (Mangalaraj and Devi, 2017). The diluted suspension of extracted RBCs (0.2 ml) was mixed with varied concentrations (1, 3, 6, 9, and 12 μg/ml) of AgNPs in PBS (0.8 ml). Diluted suspension of RBCs mixed with 0.8 ml PBS and 0.8 ml double distilled water were used as negative and positive control, respectively. The mixture was gently vortexed and incubated at room temperature for 3 h. After centrifugation (1600 rpm, 5 min) of the incubated mixture, absorbance of the supernatant was recorded at 541 nm by UV-Vis spectrophotometer (Agilent Cary 100 UV-Vis, Germany). Finally, hemocompatibility was evaluated in terms of percent hemolysis using the formula: (A_S_ - A_N_)/(A_P_ - A_N_) × 100; where “A_S_” is the sample absorbance, “A_N_” is the absorbance of negative control and “A_P_” is the absorbance of positive control (Jana et al., 2016).

#### Determining Effect of CMT-Capped Ag-MCNP on Mammalian Cells

The cytotoxic effect of polysaccharide capped nanoparticles against Mouse macrophage (RAW 264.7) cells were evaluated by MTT [3-(4,5-Dimethylthiazol-2-Yl)-2,5-Diphenyltetrazolium Bromide] assay. Approximately, 1 × 10^4^ cells were seeded in 96-well plates (Tarsons, India) and were incubated for 24 h at 37°C in a 5% CO_2_ incubator with different concentration of Ag-MCNP’s (1, 2, 3, 4, and 6 μg/ml, respectively). Raw cells seeded without nanoparticles were used as control. To determine the cell viability, MTT dye (100 μl from 0.1 mg/ml stock) was added to each well and incubated for 4 h at 37°C, 5% CO_2_ in dark. In this assay, metabolically active cells reduce the MTT salt into water insoluble purple MTT formazan crystal by mitochondrial dehydrogenase. The formazan crystals formed as a result of cellular reduction of MTT were dissolved in buffer solution (4 g NP-40 detergent in 50 ml 0.02 M HCl and 50 ml isopropanol) and incubated for 1 h at 37°C and absorbance was measured at 570 nm in an ELISA reader (BioTek, Germany). The percentage of cell viability at different doses of Ag-MCNPs was obtained by the following formula: % cell viability = [OD sample – OD control] – 100/OD control.

### Determination of Minimum Inhibitory Concentration (MIC) Breakpoints for Nanoparticles

Minimum inhibitory concentration (MIC) values were determined for both the nanoparticles *viz.* silver nanoparticle (AgNP) and silver-metal-carbohydrate nanoparticles (Ag-MCNP’s) by macrobroth double dilution method based on the guidelines of Clinical Laboratory Standard Institute (CLSI) for Enterobacteriaceae (CLSI, 2016). Ten microliters of mid log bacterial cultures (OD_600 nm_: 0.6–0.8) were inoculated into MHB with different concentration (μg/ml) of nanoparticles. Inoculated tubes were incubated at 37°C, 220 rpm in bacteriological incubator shaker (New Brunswick, NJ, United States). MIC break point values (in μg/ml) were noted after overnight incubation at the above-mentioned conditions. Further, we checked survival of the bacteria by dilution plating onto MHA plates to enumerate viable colonies.

### Determining Effect of Silver Nanoparticles on AcrAB-TolC Efflux Pump Protein Expression

The bacterial cells of (OD_600 nm_: 0.6–0.8), were treated with sub-MIC concentration of nanoparticles, i.e., AgNPs (6 μg/ml) and Ag-MCNPs (0.75 μg/ml), and incubated for specified time points (e.g., 0, 30, 60, 120, 180 min). Both the treated and control bacterial cells were pelleted down, washed once with 1X PBS (pH 7.4), dissolved in 1X PBS and kept on ice. The protein samples were preserved by adding proteinase-K, lysed by treating with 5X Lamelli buffer and heat denatured at 95°C for 5 min. Extracted protein samples were then loaded onto 12% polyacrylamide gels, transferred to polyvinylidene fluoride (PVDF) membrane (Millipore, United States) for 1 h at 17 V, blocked by 5% skimmed milk in Tris buffered Saline with 0.05% Tween-20 (1X TBST). Blots were incubated with custom synthesized anti-rabbit polyclonal anti-AcrA, anti-AcrB, and anti-TolC primary antibodies (GenScript, United States) at 1:1000 dilutions overnight at 4°C. After incubation, blots were washed thrice with 1X TBST. Further blots were incubated with goat anti-rabbit immunoglobulin secondary antibodies (1:10,000) conjugated to horseradish peroxidase. Specific bands were visualized by Chemiluminescence method using SuperSignal^TM^ West Femto maximum sensitivity substrate kit (Thermo Scientific, United States). The images were acquired using ChemiDoc and analyzed using Quantity-one software (Bio-Rad, United States).

## Results

### Resistance Profile of *Enterobacter* Isolates

The wild type *E. cloacae* clinical isolate (EspIMS6) exhibited extreme drug resistant (XDR) phenotype with resistance to β-lactams, cephalosporins belonging to second and third-generation, imipenem, fluoroquinolones (**Table 1**). The type strain, *E. cloacae* ATCC 13047 displayed MDR phenotype. Both of these isolates harbor multidrug efflux system AcrAB-TolC belonging to RND superfamily (data not shown).

**Table 1:** Antibiotic susceptibility profile of clinical *Enterobacter cloacae* isolates.

Strains	Resistant (R)	Susceptible (S)	Intermediate (I)
EspIMS6	CZX, CX, LE, CTR, TCC, PIT, GAT, A/S, CTX, AMC, OF, CIP, AMP, CXM, IPM, CPM, COT, CAZ, CAC, AT	GEN, AK	MRP
ATCC 13047	AMC, CXM, AMP, CAZ, CX, CAC, A/S	COT, CPM, IPM, CIP, GEN, GAT, AT, OF, AK, MRP, PIT, CZX, LE	CTR, CTX

*Antibiotic resistance profile of EspIMS6 and *E. cloacae* type strain ATCC 13047 control using DoDeca Enterobacteriaceae disks 1 and 2 (Himedia, India). Disk 1: Ampicillin-AMP (10 mcg), Gentamicin-GEN (10 mcg), Amikacin-AK (30 mcg), Ciprofloxacin-CIP (5 mcg), Ofloxacin-OF (5 mcg), Co-Trimoxazole-COT (25 mcg), Amoxyclav-AMC (30 mcg), Cefuroxime-CXM (30 mcg), Ceftazidime-CAZ (30 mcg), Ceftazidime/Clavulanic acid-CAC (30/10 mcg), Cefepime-CPM (30 mcg), Imipenem-IPM (10 mcg). Disk 2: Cefotaxime-CTX (30 mcg), Cetriaxone-CTR (30 mcg), Cefoxitin-CX (30 mcg), Meropenem-MRP (10 mcg), Piperacillin/Tazobactam-PIT (100/10 mcg), Aztreonam-AT (30 mcg), Gatifloxacin-GAT (5 mcg), Ampicillin/Sulbactam-A/S (10/10 mcg), Cefoperazone-CPZ (75 mcg), Levofloxacin-LE (5 mcg), Ceftizoxime-CZX (30 mcg), Ticarcillin/Clavulanic acid-TCC (75/10 mcg).*

### Characterization of Silver Nanoparticles Used

#### Physical Characterization of the Nanoparticles Used

**FIGURE 1:**
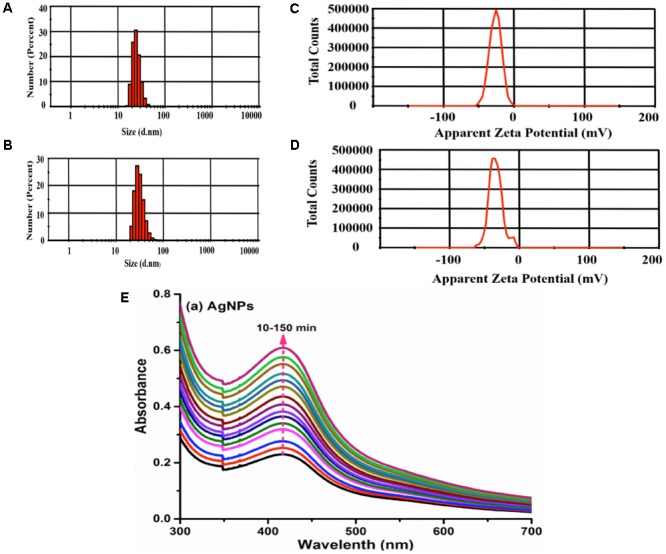
Characterization of silver nanoparticles. Size of AgNps was determined by photon correlation spectroscopy immediately after synthesis **(A)** and 6 months post-synthesis **(B)**. Stability of AgNPs in terms of Zeta potential (–34 mV) was measured immediately after synthesis **(C)** and 6 months post-synthesis **(D)**. Determination of stability of AgNPs by SPR was conducted at λmax 420 over 10–150 min **(E)**.

The size of nanoparticles has direct relevance with the stability, surface charge, bio distribution, cellular uptake, and drug release of nanomedicine (Lamprecht et al., 2001). The average size measured from photon correlation spectroscopy was found to be 140 d.nm in terms of number percent distribution (**Figures 1A,B**). The poly-dispersity index (PDI) of 0.208 indicated the monodispersed pattern of nanoparticles (Chandni et al., 2013). The surface charge of nanoparticles is another vital physical characteristic affecting its stability. The surface characteristics of polysaccharide capped AgNPs was measured upon immediate synthesis and 6 months post-synthesis. The mean zeta potential of AgNP was measured to be -34.5 ± 2.2 mV (**Figures 1C,D**). Nanoparticles with zeta potential values outside the range of +30 to -30 mV are considered stable for a longer period in suspended state (Yue et al., 2008). To validate long-term stability of suspended AgNPs, hydrodynamic diameter, PDI and surface charge were measured 6 months post-synthesis by photon correlation spectroscopy and results compared with those obtained for initially synthesized AgNPs. Our data suggest that there is slight increase in hydrodynamic diameter (161 d.nm), with PDI (0.426) and surface charge (-28.6) (**Figures 1A,B**). However, PDI smaller than 0.5 suggested that there is no agglomeration of nanoparticles and thus these particles are stable at room temperature for longer period of time (Yue et al., 2008). Further CMT-capped AgNP was characterized by SPR observed from UV-Vis spectral analysis. The occurrence of single maximum peak at wavelength 420 nm corresponded to the SPR of AgNPs (**Figure 1E**), and reflected about the size of AgNPs to be around 30–40 nm (Oldenburg, 2012). Further characterization of this AgNP such as size, dispersion, crystallinity and presence of silver has been performed by TEM, FE-SEM, XRD and EDAX methods, which was reported in our previous article (Sanyasi et al., 2016).

#### Hemocompatibility of Silver Nanoparticles

**FIGURE 2:**
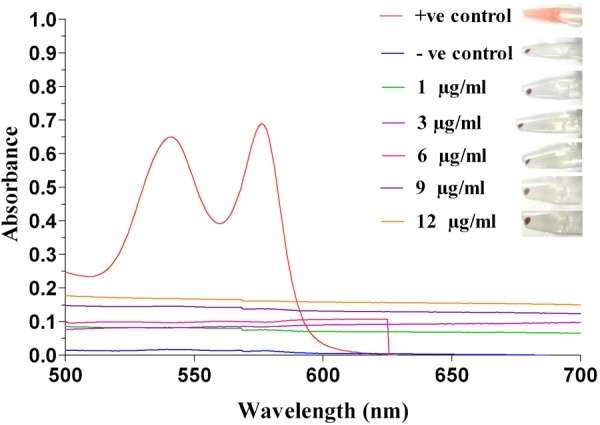
Hemolysis activity of silver nanoparticle. Determination of hemolytic activity at different concentrations (1, 3, 6, 9, and 12 μg/ml) of Ag-NPs, along with positive and negative control, recorded at 541 nm by UV-Vis spectrophotometer.

Extent of hemolysis of red blood cells was determined to check hemocompatibility of silver nanoparticles. It was observed that AgNPs at concentrations of 1, 3 and 6 μg/ml, exhibited only 0.063, 0.31, and 0.63% of hemolysis, respectively (**Figure 2**). At higher concentrations of AgNP (i.e., 9 and 12 μg/ml) hemolysis was approximately 2%. Nanoparticles exhibiting hemolysis below 5% are considered hemocompatible (Jana et al., 2016). Hence, our results indicated that AgNPs to be hemocompatible.

#### Effect of Ag-MCNPs on Cell Viability

**FIGURE 3:**
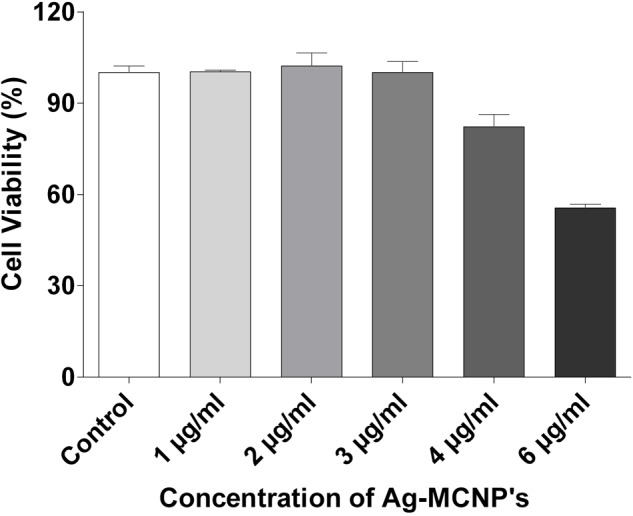
Cytotoxicity assay of silver nanoparticle. Effect of Ag-MCNP on cell viability was determined by MTT assay with RAW 264.7 cells with increasing concentrations (1, 2, 3, 4, and 6 μg/ml) of the nanoparticle. Percentage of cell viability was calculated with respect to untreated cells.

We had previously shown that AgNP’s were non-toxic to mammalian cells (Sanyasi et al., 2016). Here, we have assessed the cytotoxic effect of Ag-MCNPs on macrophage RAW 264.7 cell line using MTT assay. The MTT assay is rapid, sensitive, and inexpensive method to determine cell viability. The dose dependent viability of RAW 264.7 cells in different concentration of the Ag-MCNPs is shown in **Figure 3**. Cell viability result revealed that 99% cells were viable in presence of Ag-MCNPs at concentration of up to 3 μg/ml, while at 4 μg/ml concentration of Ag-MCNPs, 82% cells remained viable (**Figure 3**). However, at higher concentration (6 μg/ml) of Ag-MCNP, cell viability was reduced to 55% suggesting that Ag-MCNP was toxic to mammalian cells at this dosage. This indicated that Ag-MCNPs below concentrations of 6 μg/ml are non-toxic to mammalian cells. Notably, the concentration of Ag-MCNPs that was antibacterial in nature (i.e., 1.5 and 3 μg/ml), are non-toxic to mammalian cells and hence could be suitable for biomedical applications.

### Antimicrobial Efficacy of AgNP and Ag-MCNP

We determined the MIC breakpoints for sliver nanoparticle (AgNP) and Ag-MCNP. We observed that both the nanoparticles tested significantly inhibited bacterial growth in a dose dependent manner (**Table 2** and Supplementary Figure S1). However, MIC breakpoint for AgNPs was 12 μg/ml for both EspIMS6 and *E. cloacae* ATCC 13047. Interestingly, Ag-MCNPs had a much lower MIC breakpoint of 1.5 and 3 μg/ml for isolates EspIMS6 and *E. cloacae* ATCC 13047, respectively. The results clearly indicated that Ag-MCNPs was more efficient in killing the MDR *E. cloacae* compared to AgNPs alone.

**Table 2:** Antibacterial activity of silver and silver-metal composite nanoparticles.

Isolates ID	MIC (in μg/ml)	
	AgNP	Ag-MCNP
EspIMS6	12	1.5
ATCC-13047	12	3

### Effect of Silver Nanoparticles on AcrAB-TolC Efflux Pump Protein Expression

As reported from the literature, success of potential antibacterial agents rested upon their efficacy to be retained in the cell vis-à-vis being effluxed out. We investigated this by studying the effect of silver nanoparticles on expression of multidrug efflux pump proteins AcrAB-TolC.

**FIGURE 4:**
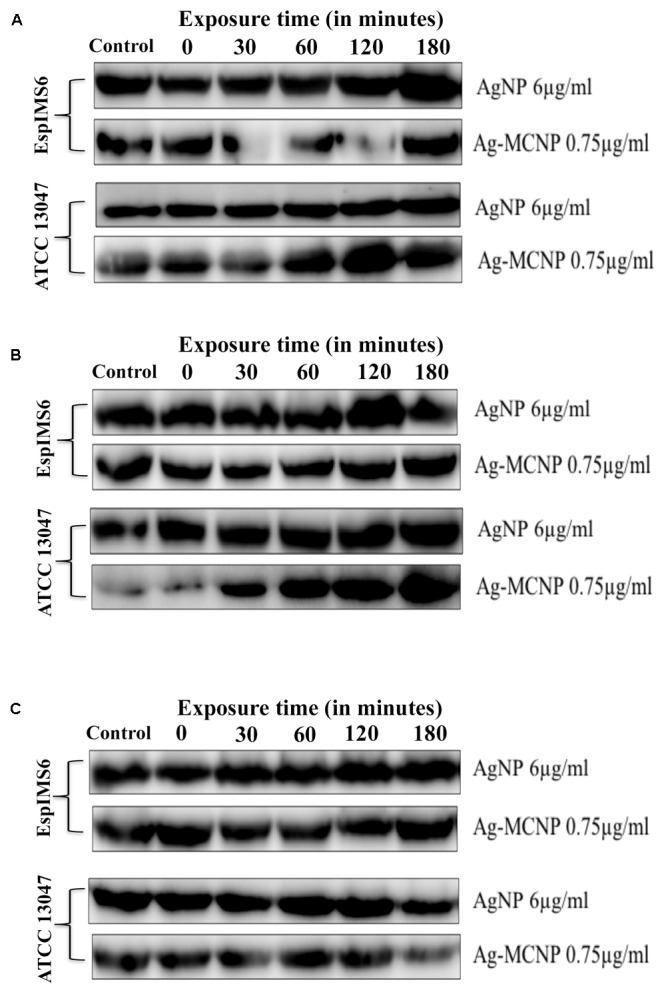
Effect of silver and silver-metal composite nanoparticles on AcrAB-TolC expression. **(A)** AcrA (42 kDa) protein expression in EspIMS6 (top panel) and *Enterobacter cloacae* ATCC 13047 (bottom panel); **(B)** AcrB (∼38 kDa) protein expression in EspIMS6 (top panel) and *E. cloacae* ATCC 13047 (bottom panel); **(C)** TolC (55 kDa) protein expression in EspIMS6 (top panel) and *E. cloacae* ATCC 13047 (bottom panel).

Overall immunoblotting results revealed that presence of silver nanoparticles did not significantly affect AcrA and TolC efflux pump protein expression (**Figures 4A,C**). However, in the *E. cloacae* ATCC 13047, AcrB, and TolC protein expression showed slight albeit insignificant decrease in presence of Ag-MCNPs (**Figures 4B,C** and Supplementary Figure S2). Inhibition of AcrB monomeric protein expression was more prominent in clinical isolate EspIMS6, where only 38 kDa AcrB protein was consistently expressed at all the time points of exposure to AgNP (at 6 μg/ml) as well as Ag-MCNP (at 0.75 μg/ml) (**Figure 4B** and Supplementary Figure S2). Though, ∼112 kDa band was less expressed in *E. cloacae* ATCC 13047 in response to AgNP (at 6 μg/ml), a 38 kDa band of AcrB was observed post 30 min of treatment with Ag-MCNP at a concentration of 0.75 μg/ml in *E. cloacae* ATCC 13047 (**Figure 4B**, panels 3 and 4 and Supplementary Figure S2). It is pertinent to mention here that, AcrB is a transmembrane RND transporter protein of the tripartite efflux pump assembly. The functional membrane embedded protein is a homotrimer with molecular masses of ∼380 kDa, with each monomer of approximately 112 kDa (Tikhonova et al., 2011). However, in both isolates EspIMS6 and *E. cloacae* ATCC 13047 treated with the AgNP nanoparticles, it was shown to inhibit monomeric 112 kDa bands, and showed bands corresponding to ∼38 kDa (Supplementary Figure S2). This ∼38 kDa band perhaps represents the truncated by product of the AcrB protein complex. It is pertinent to mention that the antibodies used were beyond doubt.

Expression of outer membrane protein TolC decreased post 120 min in *E. cloacae* ATCC 13047, in presence both the silver nanoparticles tested *viz.* AgNPs and Ag-MCNPs (**Figure 4C**, panels 3 and 4). Here too we did not observe any significant changes in TolC expression for the wild type isolate EspIMS6 in presence of either of the nanoparticles (**Figure 4C**, panels 1 and 2).

## Discussion

The present study reports no decrease in antibacterial efficacy of silver nanoparticles *viz.* AgNP and Ag-MCNP, despite expression of AcrAB-TolC efflux protein in MDR bacteria. This finding is significant as it reports no noticeable effect of nanoparticles on AcrAB-TolC efflux pump protein expression that are implicated in MDR phenotype. Secondly, the results corroborate with our previous report and confirm sustainability of using the said silver nanoparticles as antibacterial agents.

During the past decade, silver nanoparticles owing to their low toxicity, antibacterial and wound healing properties, have emerged as promising therapeutic alternatives (Ambrožová et al., 2017). To ensure feasibility of large-scale use of nanoparticles for medicinal purposes, eco-friendly approaches have been widely adapted to generate silver nanoparticles. Such approaches circumvent the use of toxic chemicals and hazardous equipment. Eco-friendly green synthesis approach utilizes natural cost-effective reductants such as microorganisms and plant products (Rao and Tang, 2017). We had earlier reported utilization of green synthesis approach to generate polysaccharide-capped AgNPs (Sanyasi et al., 2016).

Few of the important physical parameters essential for development of nanoparticles include size and surface charge that affects their stability (Lamprecht et al., 2001). In the present study, photon correlation spectroscopy results revealed that the CMT-capped AgNPs were smaller in size and are monodispersed in nature (Chandni et al., 2013). The mean zeta potential of AgNPs used was measured to be -34.5 ± 2.2 mV, suggesting higher electrical charge on their surface and thus, approved their long-term stability (Yue et al., 2008). SPR characterization of AgNPs demonstrated their size to be around 30–40 nm, which corroborated well with previous report (Oldenburg, 2012). Biocompatibility is extremely important feature for nanoparticles to be used as therapeutic agents. For this, we performed hemolysis assay that described the AgNPs to be non-hemolytic. We had previously reported AgNPs to be less cytotoxic toward mammalian cells even at higher concentrations (Sanyasi et al., 2016). The newly developed Ag-MCNPs reported here, too displayed minimal cytotoxic activity toward mammalian cells. Both AgNPs and Ag-MCNPs revealed excellent antimicrobial activity against multiple drug resistant *E. cloacae* isolates at low dose. Our results corroborated well with previous reports suggesting antimicrobial properties of silver nanoparticles (Kim et al., 2007; Chen et al., 2014), that boosted their candidature as potential antimicrobial agents. Durán et al. (2016) proposed cell membrane disruption by the silver ions as a plausible mechanism of antibacterial activity by silver nanoparticles.

However, one of the less addressed aspect has been the effect of AgNPs on bacterial efflux systems. Kovács et al. (2016) reported AgNPs to inhibit activity of *p*-glycoproteins efflux pump (belonging to ABC transporter family) in MDR cancer cells, thereby enhancing effectiveness of chemotherapy. Christena et al. (2015) have established the efflux inhibitory properties of copper nanoparticles (CuNPs) in tackling MDR *Staphylococcus aureus* and *Pseudomonas aeruginosa* isolates. However, effect of AgNPs on expression of bacterial MDR efflux pumps has not been elucidated. AcrAB-TolC efflux pumps have been found to mediate MDR phenotype in many clinically significant gram-negative pathogens including *E. cloacae* (Piddock, 2006; Pérez et al., 2012; Amaral et al., 2013). These efflux systems efficiently extrude out of the bacterial cell, structurally divergent classes of drugs, resulting in multiple antibiotic resistance phenotype (Poole, 2005; Sun et al., 2014). This prompted us to study the effect of AgNPs and Ag-MCNPs on AcrAB-TolC efflux pump protein expression. Our results indicated that AgNPs affected the expression of AcrB transporter protein to a greater extent as compared to AcrA and TolC. Functional AcrB is a trimeric RND transporter protein with each monomer of ∼112 kDa (Du et al., 2015). Review of literature reveals antimicrobial drugs to induce over expression of AcrAB-TolC efflux pump protein, which in turn facilitates their rapid extrusion (Rosenberg et al., 2003). In the present study, expression of functional AcrB protein was reduced in *E. cloacae* isolates upon addition of silver nanoparticles (Supplementary Figure S2). It is pertinent to mention that AcrB protein is responsible for substrate recognition and binding of diverse structures (Sun et al., 2014). The results provide circumstantial evidence indicative of AgNPs functioning as efflux inhibitor. Efflux inhibitory role of metal nanoparticles have been hypothesized to be either by direct binding to active site of the efflux pumps or hindering efflux kinetics (Gupta et al., 2017). However, owing to the small size of nanoparticles and substrate non-specific nature of efflux pumps proteins these explicit mechanisms still remain elusive. Earlier studies have shown that expression of AcrB transporter is affected by cellular metabolites (Ruiz and Levy, 2014) and hence forms an ideal target for efflux and virulence inhibitor design (Blair et al., 2015). Also, AcrB is a proton motive force (PMF) dependent RND transporter. Interestingly, Dibrov et al. (2002) in a study on mechanism of action of nanoparticles, observed that metal nanoparticles such as AgNPs, dissipate PMF by interfering with bacterial respiration. Hence, energy required for this tripartite efflux system may be hindered by the addition of AgNPs. In the present study, in presence of AgNPs, though TolC and AcrA protein expression remained more or less unaffected, the absence of a functional monomeric AcrB perhaps renders the bacterial cells impaired for efflux activity. Change in efflux activity in *Enterobacter* spp. by AgNPs has been reported in context of EmmDR efflux pump [belonging to multiple antibiotic and toxic extrusion (MATE) superfamily] (Gui et al., 2013). However, to the best of our knowledge, this is the first report on effect of silver nanoparticles on AcrAB-TolC efflux protein expression. This study indicates potential efflux inhibitor property of the AgNPs.

To summarize, the reported silver nanoparticles generated by green synthesis method, are highly stable, small in size, non-hemolytic with minimal cytotoxic effect. Further, their anti-microbial activity is not affected by expression of AcrAB-TolC efflux proteins. Such versatile repertoire of qualities enables us to report the said silver nanoparticles as strong candidates as antibacterial agents. The results of the present study support further development of silver nanoparticle formulations as alternate therapeutic agents to combat infections caused by MDR gram-negative Enterobacterial pathogens.

## Author Contributions

The present study was conceived by HM and executed by MM. SK, LG, RM, and CG developed and characterized the nanoparticles.

## Conflict of Interest Statement

The authors declare that the research was conducted in the absence of any commercial or financial relationships that could be construed as a potential conflict of interest. The reviewer RP and handling Editor declared their shared affiliation.
