# Supplementary material for: Antibacterial Efficacy of Polysaccharide Capped Silver Nanoparticles Is Not Compromised by AcrAB-TolC Efflux Pump
Source: Front Microbiol. 2018 May 4;9:823. doi: 10.3389/fmicb.2018.00823 (PMC5945830; doi:10.3389/fmicb.2018.00823)
Supplement: Supplementary file 1 [file Data_Sheet_1.pdf]

SUPPLEMENTARY MATERIAL

TITLE: Antibacterial efficacy of polysaccharide capped silver nanoparticles is not affected by AcrAB-TolC efflux pump

Mitali Mishra[1], Satish Kumar[2], Rakesh K. Majhi[1], Luna Goswami[2], Chandan Goswami[1], Harapriya Mohapatra[1]*

[1]School of Biological Sciences, National Institute of Science Education & Research, HBNI, Jatni - 752050, India.

[2]School of Biotechnology, KIIT University, Patia, Bhubaneswar - 751024, India.

*Correspondence: hm@niser.ac.in, hmsbsniser@gmail.com

Figure S1: Effect of silver and silver-metal composite nanoparticles on bacterial growth

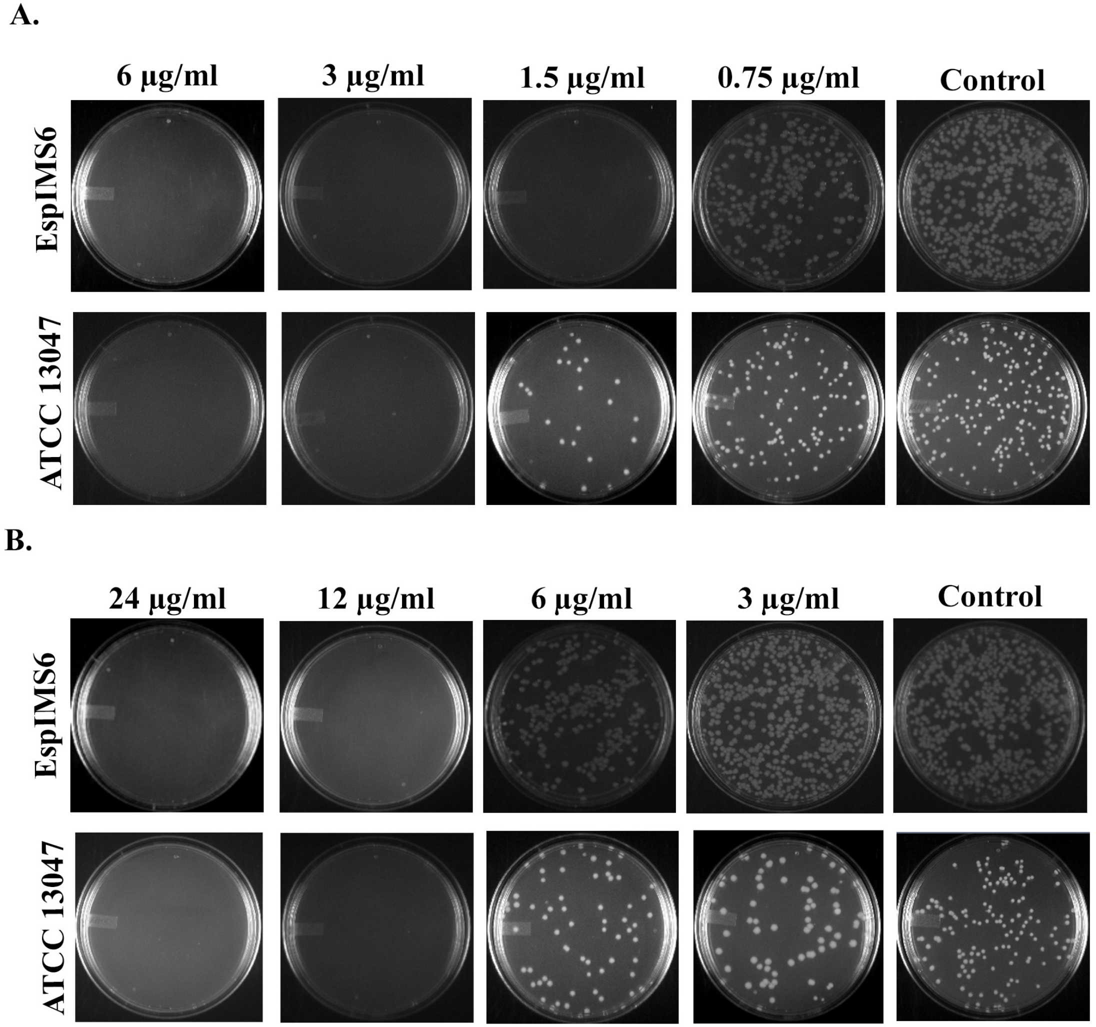

Antibacterial properties of sliver-metal composite (Ag-MCNPs- Panel A) and silver (AgNP-Panel B) nanoparticles on bacterial growth was determined by spread plating onto MHA plates after MIC. Images were recorded using gel documentation system (Biorad, USA).

Figure S2: Whole blot showing the presence of monomeric AcrB protein (~112 kDa in size) in presence of nanoparticles AgNP and Ag-MCNPs in *E.cloacae* isolates.

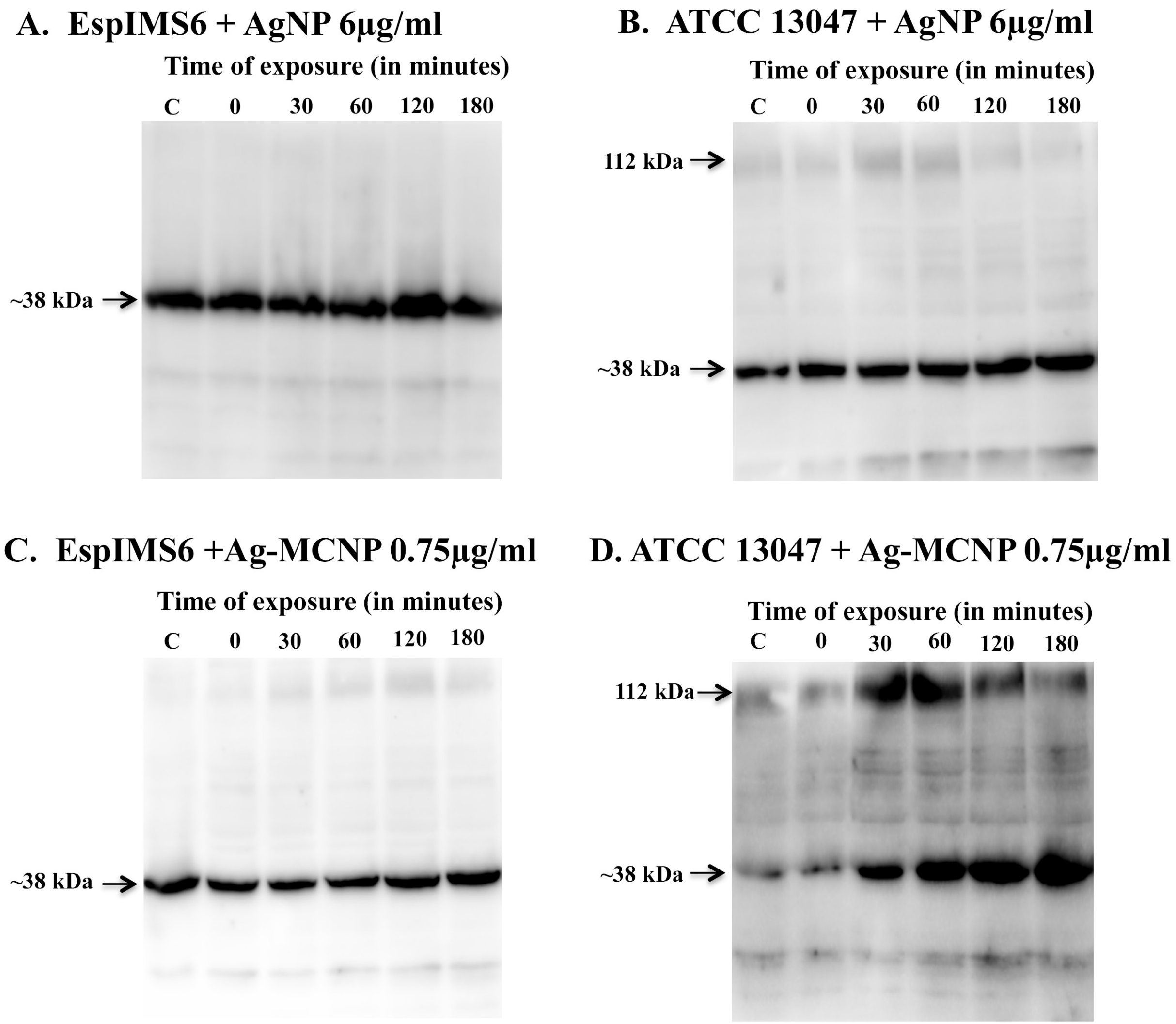

The whole immunoblot showed a ~38 kDa AcrB protein expression in response to sub lethal concentrations of AgNP (6µg/ml) and Ag-MCNP (0.75µg/ml) in EspIMS6 (A, C) and ATCC 13047 (B, D).
